# Networks of care to strengthen primary healthcare in resource constrained settings

**DOI:** 10.1136/bmj-2022-071833

**Published:** 2023-03-13

**Authors:** Enoch Oti Agyekum, Katherine Kalaris, Blerta Maliqi, Allisyn C Moran, Andrews Ayim, Sanam Roder-DeWan

**Affiliations:** 1 World Bank Group, Health Nutrition and Population, Country Office, Accra, Ghana; 2 Health Systems Collaborative, University of Oxford, Oxford, UK; 3 Department of Maternal, Newborn, Child, Adolescent Health and Ageing, World Health Organization, Geneva, Switzerland; 4 Ghana Health Service, Accra, Ghana; 5 World Bank Group, Health Nutrition and Population, Global Practice, Washington, DC, USA; 6 Dartmouth Medical School, Hanover, NH, USA

## Abstract

Networks of care are a promising way to provide support and resources for isolated primary care workers and deserve more research, argue **Enoch Oti Agyekum and colleagues**

High quality primary healthcare is a cornerstone of country strategies to achieve universal health coverage but has been difficult to implement and scale. Systemic challenges such as lack of coordination and an inadequate health workforce limit the reach and impact of primary healthcare, which should be able to deliver 90% of essential interventions included in universal coverage.^1^ ^2^ Pandemic related setbacks in progress towards universal health coverage makes this a particularly important time to reinvigorate primary care.

Primary care in most low income health systems is underfunded and understaffed,^3^ especially in rural areas, leaving healthcare staff working alone to respond to their community’s needs. By 2030 the global workforce shortage is projected to be over 10 million. Although this figure is lower than previous estimates, the distribution is inequitable.^4^ ^5^ Two thirds of the projected shortage will be in the most resource constrained settings, with half in the World Health Organization’s Africa region alone. Primary healthcare workers in this region already report feeling isolated and unsupported by the health system.^6^

Working alone means evidence based approaches to improving quality such as group based problem solving are impossible.^7^ Rural primary care facilities in resource constrained settings may also experience low patient volumes. Greater patient volume, if managed appropriately, is correlated with improved quality of care^8^ because healthcare workers have opportunities to build expertise and confidence, keep skills up to date, and remain alert to signs that a patient needs referral to higher level care.^9^

Though the workforce is often spread thin, the wide availability of mobile phones, improved roads, and transport systems are connecting healthcare workers—and the facilities they work in—in new ways. Against this background, interest in more formal collaboration between facilities in the form of networks of care is growing. Ghana, for example, is expanding the use of networks of care to provide primary care and has received $181m (£150m; €170m) in support for this from the World Bank and the Global Financing Facility.^10^ In Côte d’Ivoire, the government has decreed that the health system should be organised into networks and is working to put this model into practice.^11^ If further research supports the effectiveness of networks, wider use could offer a systems level opportunity to overcome the challenges associated with providing quality primary healthcare in resource constrained health systems through collaborative learning, resource sharing, and mutual support.

## Defining networks of care

**Fig 1:**
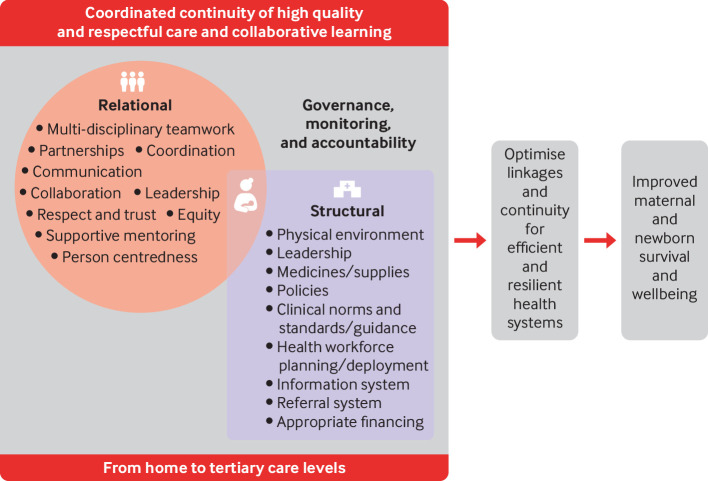
Key components of a network of care: structural and relational elements, goals, and potential effect on maternal and newborn health. Adapted from a figure by Kalaris et al^13^

Though collaboration among care facilities is not new, networks of care first appeared in the global health literature as a discrete concept in 2020. They were defined as “a group of public and/or private health service delivery sites deliberately interconnected through an administrative and clinical management model which promotes a structure and culture that prioritizes client-centered, effective, efficient operation and collaborative learning, enabling providers across all levels of care, not excluding the community, to work in teams and share responsibility for health outcomes.”^12^ Building on this definition, a landscape review led by WHO, identified key structural and relational elements of networks working to improve maternal and newborn healthcare (fig 1).^13^

Networks of care share characteristics with other approaches that target the organisation of a health system to improve performance. For example, networks may be nested within district health systems but also include links to tertiary care facilities or may cross administrative boundaries. Unlike other approaches to reorganisation such as service delivery redesign,^14^ however, the formation of networks of care does not necessarily mean changing where services are provided. Although structural processes are important, the focus is on building relations and collaboration between service providers. 

## Case studies suggest improved health outcomes

Evidence on the impact of networks of care on health outcomes is limited and focuses largely on maternal, newborn, and child health outcomes.^12^ An evaluation of the effect of introducing a network of care in northern Nigeria, for example, found maternal deaths fell by 37% (P≤0.0003), neonatal deaths by 43% (P<0.0001), perinatal deaths by 27% (P<0.0001), and stillbirths by 15% (P=0.0018) 18 months after implementation.^15^ The networks used a client centred approach to service delivery, integrated traditional birth attendants, improved operational standards and transport for emergency referral, revitalised community data collection, supported procurement and supply chain, strengthened monitoring, and improved clinical skills through training and mentoring programmes.^16^ An evaluation of a network of care in Northern Province, Zambia, also reported significant decreases of 41%, 45%, and 43% in maternal, neonatal, and perinatal mortality rates, respectively, between 2019 and programme end in 2021 with similar activities to those implemented in northern Nigeria.^17^

A non-randomised quasi-experimental evaluation^18^ ^19^ ^20^ of the expanding maternal and neonatal survival programme in Indonesia similarly found that the case fatality rate among pregnant women with complications admitted to a network of care hospitals decreased by 50% (on average from 5.4 to 2.6 deaths per 1000 cases of obstetric complications), and very early neonatal mortality decreased by 21% over the 21-45 months of the project.^18^ ^21^ This network focused on peer-to-peer mentoring to improve facility performance, facility based reviews of maternal and newborn deaths, maintaining healthcare worker skills through emergency drills, using data for decision making, strengthening the performance standards, organisation, and communication of referrals, deployment of community motivators, and civic engagement.^21^

In rural Madagascar, a district focused network strengthened primary care by improving patient referral and providing greater availability and readiness of services at different levels of care. The network also enhanced communication and supervision to ensure clinical quality, provided social support to patients, and grew community engagement.^22^ A longitudinal cohort study on women and children in the district using this network of care found a 12.6% fall in infant mortality and a 36% fall in neonatal mortality, while the rest of the district (not part of the network) saw no changes. However, the fall in mortality for children under 5 years was similar within (19%) and outside the network (14.9%).^23^ The coverage index for maternal, newborn, and child healthcare increased from 49.3% to 64.1% in the network of care but barely increased (43.2% to 44.9%) in the rest of the district. Curative interventions for children under 5 increased by 20% in the network but did not improve or worsened in the rest of the district.^23^ Additionally, a 51% increase in care seeking among children with reported diarrhoea, fever, or persistent coughing was reported over two years in the network, compared with a 7% decrease outside (P=0.01).^24^ As one strategy to reduce inequities in access to care, the network introduced fee exemptions for targeted medicines and services and found that the use of services increased by 65% for all patients, 52% for children under 5, and over 25% for maternity consultations.^25^

Networks of care have not yet been evaluated using experimental designs with robust controls. The complex nature of health system interventions makes such studies challenging, but the available case studies do suggest that these networks improve the quality of healthcare and health outcomes and justify future studies, particularly on their potential to reduce inequities in access to care.

## Role of strong relationships

The mechanisms through which networks of care promote and support strong relationships in healthcare and how these might lead to better outcomes both need further investigation. Trust, teamwork, collaboration, communication, respect, and leadership feature strongly in many of the documented networks, though each mobilises the relational elements in different ways. For example, a sense of purpose and confidence derived from the collaborative formation of a public-private sector network in Dar es Salaam, Tanzania, is believed to have motivated senior healthcare workers to check-in with colleagues to offer assistance in providing care. A case study suggests that the check-ins improved teamwork, which facilitated a decline in hospital maternal and perinatal deaths.^26^

The network in Metro Manila, Philippines, is credited with transforming the professional culture and creating trust between clinicians at a tertiary public sector hospital and public and private midwifery clinics. Qualitative data show that previous mistrust between these healthcare workers was allayed when the network leader began visiting midwifery clinics in the catchment area to meet the midwives and saw that they provided good care. This change in trust spread to other healthcare workers at the hospital and facilitated the development of relationships between providers across the network. With these connections in place, hospital providers felt comfortable referring women with low risk pregnancies to the midwifery clinics, allowing the tertiary hospital to use its time and resources for more complex cases. This approach became particularly important during the covid-19 pandemic, when referrals from the tertiary hospital emergency department to midwifery clinics increased by 3.5% compared with pre-pandemic referrals.^27^

Although networks of care aim to build relationships, they also present challenges. These include inefficient coordination^21^ and communication,^16^ ^21^ ^27^ reluctance from hospital management to support networks of care,^27^ lack of trust between providers across different levels and sectors of care, and conflicts of professional culture.^27^ During the formation of the network in Metro Manila, hospital management at the tertiary hospital were initially hesitant to support the creation of the network because of a fear of losing income and uncertainty about the quality of care at other sites in the network.^27^ Networks also face structural challenges such as lack of transportation and communication infrastructure, overcrowding at higher level facilities,^28^ workforce shortages,^26^ a reliance on paper records, and a need for facility level quality improvement initiatives.^27^ For example, the ability of the Dar es Salaam network to provide high quality care was affected by a shortage of critical staff resulting from redundancy of healthcare workers and workforce transition out of clinical care.^26^

## Need for implementation research

The process of implementing and scaling networks of care needs to be systematically documented and studied to establish the effectiveness, barriers, and effects of these models of service delivery. Implementation research, ideally embedded in government rather than in outside projects, can shed light on these processes. For example, the rollout of primary care networks in Ghana is accompanied by a robust implementation research programme led by the government. Under the networks sub-district health systems link community based health planning and primary healthcare services to health centres, acting as hubs with regular joint review meetings. Work to expand the Ghanaian model of networks is intended to complement and strengthen the sub-district and district health systems by building relationships that allow for better shared care of patients, resource distribution, and knowledge sharing. The implementation research allows for regional level adaptation every six months. A pilot showed that facilities went from competing with each other to collaborating and working together to extend services to their communities.^29^

The available literature in this nascent area of study suggests that networks of care have the potential to reinforce the tenets of primary healthcare and may improve maternal, newborn, and child health outcomes in resource constrained settings. The focus on relational elements, such as trust, communication, and teamwork, when implemented well and in conjunction with support for structural elements, is likely to contribute to improvements. Qualitative methods will be essential for understanding how networks function, and pragmatic evaluation approaches will be needed to assess this type of complex intervention. As countries design, pilot, and rollout networks in diverse and contextually appropriate ways, robust research to understand the process and impact are necessary to contribute to global learning on this novel approach to health system organisation.

**Key messages**

- Collaboration between healthcare facilities, in the form of networks of care has the potential to improve efficiency, quality, and outcomes in resource constrained settings
- Case studies of successful networks of care signal that the approach can strengthen structural and, most importantly, relational elements among facilities
- Networks of care are being implemented at primary healthcare level in many countries, providing opportunities for further research on its effects

## Web extra

BMJ Infographic

Infographic: Networks of care

## Notes

Contributors and sources: This article was conceived as part of professional discussions on the networks of care service delivery approach. EOA coordinated the ideation process and developed the first draft based on a note prepared by SR. KK contributed to the writing of the paper and shaping of the argument. All authors, contributed to the development of the paper’s core argument and writing of the article. EOA and KK are the first authors and guarantors. The views are those of the authors and do not necessarily represent the views, decisions, or policies of the institutions with which they are affiliated.

Provenance and peer review: Commissioned; externally peer reviewed.

This article is part of a collection proposed by the World Health Organization and the World Bank and commissioned by *The BMJ*. *The BMJ* peer reviewed, edited, and made the decision to publish these articles. Article handling fees are funded by the Bill and Melinda Gates Foundation. Jennifer Rasanathan, Juan Franco, and Emma Veitch edited this collection for *The BMJ*. Regina Kamoga was the patient editor.
