# Supplementary material for: Networks of care to strengthen primary healthcare in resource constrained settings
Source: BMJ. 2023 Mar 13;380:e071833. doi: 10.1136/bmj-2022-071833 (PMC9999466; doi:10.1136/bmj-2022-071833)
Supplement: Supplementary file 1 — Infographic: Networks of care [file agye071833.wf1.pdf]

thebmj Visual summary

Networks of care

Worldwide achievements in recent years

Interest is growing in the potential for collaboration between facilities in the form of networks of care to improve healthcare quality. While evidence of the impact of networks of care on health outcomes is limited, networks of care have been implemented in countries around the world with promising results for maternal, newborn and child health outcomes. More preliminary evidence points to positive impacts of networks of care on quality in primary healthcare. This graphic summarises eight examples of networks of care, highlighting some of their features and associated health outcomes.

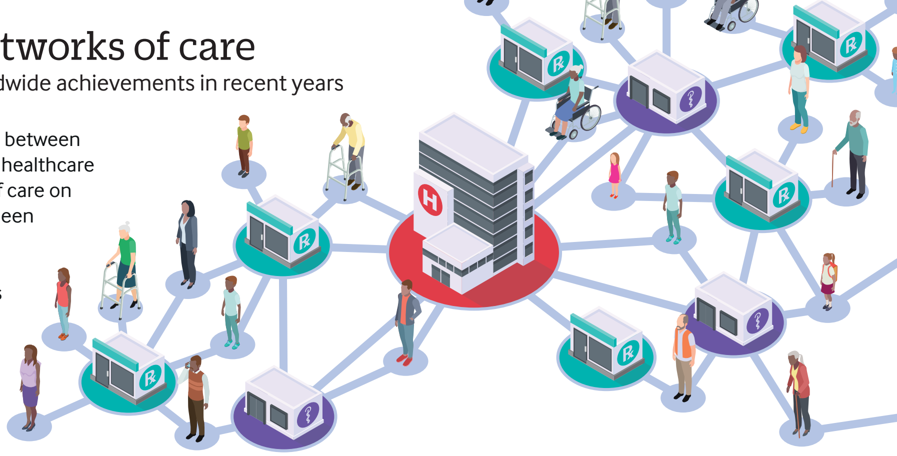

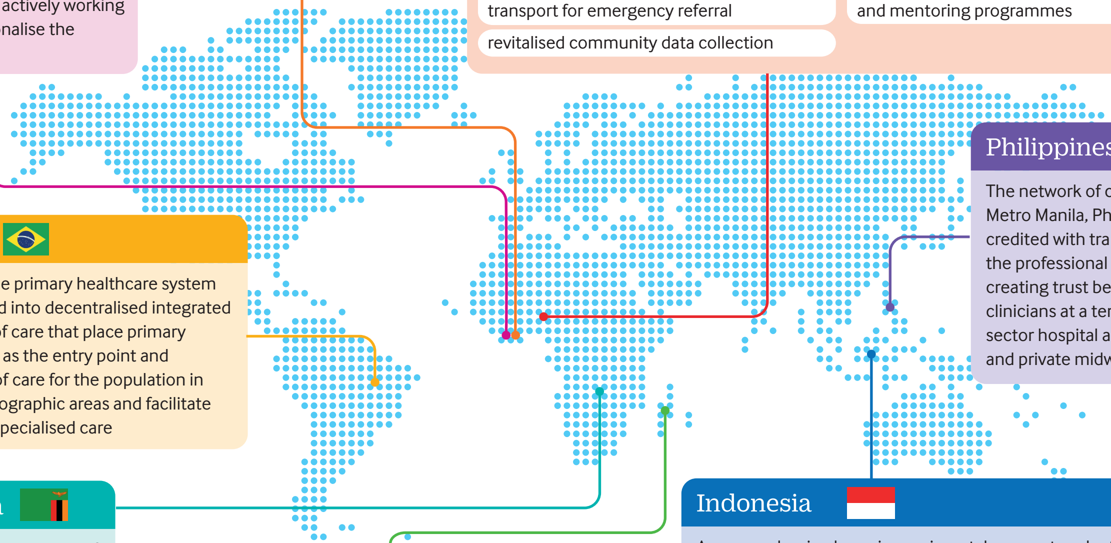

Côte d'Ivoire

In Côte d'Ivoire, the government set a decree for the health system to be organised into networks of care and is actively working to operationalise the concept

Ghana

The government of Ghana is scaling networks of care as a service delivery approach to providing primary healthcare and has received US$181 million from the World Bank and the Global Financing Facility in support of this

Nigeria

A pre-post evaluation of a network of care in northern Nigeria documented statistically significant reductions of 37% in maternal deaths, 43% in neonatal deaths, 27% in perinatal deaths, and 15% in stillbirths in 18 months (baseline 7-9 months, endline 16-18 months). It featured:

- a client centred approach to service delivery
- supported procurement and supply chain
- integrated traditional birth attendants
- strengthened monitoring
- improved operational standards and transport for emergency referral
- improved clinical skills through training and mentoring programmes
- revitalised community data collection

Philippines

The network of care in Metro Manila, Philippines, is credited with transforming the professional culture and creating trust between clinicians at a tertiary public sector hospital and public and private midwifery clinics

Brazil

In Brazil, the primary healthcare system has evolved into decentralised integrated networks of care that place primary healthcare as the entry point and organiser of care for the population in defined geographic areas and facilitate access to specialised care

Zambia

A pre-post evaluation of a network of care in Northern Province, Zambia, published in 2022 reported statistically significant decreases of 41%, 45%, and 43% in maternal, neonatal, and perinatal mortality rates, respectively

Madagascar

A longitudinal cohort study on women and children in a district in rural Madagascar using a network of care in primary healthcare found a 19% decline in under-5 mortality, a 12.6% decline in infant mortality, and a 36% decline in neonatal mortality, while the rest of the district (not part of the network of care) saw similar declines in under-5 mortality (14.9%) but no changes in infant or newborn mortality. The network of care featured:

- improved patient referral
- social support for patients
- greater availability and readiness of services at different levels of care
- enhanced communication and supervision to ensure clinical quality
- growth of community engagement

Indonesia

A non-randomised quasi-experimental pre-post evaluation of the Expanding Maternal and Neonatal Survival programme in Indonesia found that the case fatality rate among pregnant women with complications admitted to hospitals in the network of care decreased by 50% (on average from 5.4 to 2.6 deaths per 1000 cases of obstetric complications), and very early neonatal mortality decreased by 21% over the project period of 21-45 months. The network of care featured:

- peer-to-peer mentoring to improve facility performance
- using data for decision making
- strengthening referral performance standards, organisation, and communication
- facility based maternal and newborn death reviews
- deployment of community motivators and civic engagement
- maintaining healthcare worker skills through emergency drills

thebmj Read the full article online https://bit.ly/bmj-noc

© 2023 BMJ Publishing Group Ltd.

See more visual summaries http://www.bmj.com/infographics

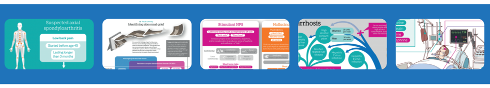

Disclaimer

Validation This infographic is not a validated clinical decision aid

Updating This information is provided without any representations, conditions, or warranties that it is accurate or up to date

Responsibility BMJ and its licensors assume no responsibility for any aspect of treatment administered with the aid of this information

Risks Any reliance placed on this information is strictly at the user's own risk

For the full disclaimer wording see BMJ's terms and conditions: http://www.bmj.com/company/legal-information/
